# Quality review of an adverse incident reporting system and root cause analysis of serious adverse surgical incidents in a teaching hospital of Scotland

**DOI:** 10.1186/1754-9493-6-21

**Published:** 2012-08-29

**Authors:** Maziar Khorsandi, Christos Skouras, Kevin Beatson, Afshin Alijani

**Affiliations:** 1 Department of Cardiothoracic Surgery, Royal Infirmary of Edinburgh, 51 Little France Crescent, Old Dalkeith road, Edinburgh, EH16 4SA, UK; 2 Department of General Surgery, Royal Infirmary of Edinburgh, 51 Little France Crescent, Old Dalkeith road, Edinburgh, EH16 4SA, UK; 3 Department of Orthopaedic Surgery, Royal Infirmary of Edinburgh, 51 Little France Crescent, Old Dalkeith road, Edinburgh, EH16 4SA, UK; 4 Department of Surgery, Ninewells Hospital, Dundee, DD1 9SY, UK

**Keywords:** Risk management, Harm reduction, Root cause analysis, Incident reporting, Adverse incidents

## Abstract

**Background:**

A significant proportion of surgical patients are unintentionally harmed during their hospital stay. Root Cause Analysis (RCA) aims to determine the aetiology of adverse incidents that lead to patient harm and produce a series of recommendations, which would minimise the risk of recurrence of similar events, if appropriately applied to clinical practice. A review of the quality of the adverse incident reporting system and the RCA of serious adverse incidents at the Department of Surgery of Ninewells hospital, in Dundee, United Kingdom was performed.

**Methods:**

The Adverse Incident Management (AIM) database of the Department of Surgery of Ninewells Hospital was retrospectively reviewed. Details of all serious (red, sentinel) incidents recorded between May 2004 and December 2009, including the RCA reports and outcomes, where applicable, were reviewed. Additional related information was gathered by interviewing the involved members of staff.

**Results:**

The total number of reported surgical incidents was 3142, of which 81 (2.58%) cases had been reported as red or sentinel. 19 of the 81 incidents (23.4%) had been inappropriately reported as red. In 31 reports (38.2%) vital information with regards to the details of the adverse incidents had not been recorded. In 12 cases (14.8%) the description of incidents was of poor quality. RCA was performed for 47 cases (58%) and only 12 cases (15%) received recommendations aiming to improve clinical practice.

**Conclusion:**

The results of our study demonstrate the need for improvement in the quality of incident reporting. There are enormous benefits to be gained by this time and resource consuming process, however appropriate staff training on the use of this system is a pre-requisite. Furthermore, sufficient support and resources are required for the implementation of RCA recommendations in clinical practice.

## Background

Patient harm is a well recognised fact; up to 10% of patients acutely admitted to hospital are unintentionally harmed [1-3], up to two thirds of which are surgical patients [4]. It is therefore imperative that measures are taken to minimise the risk of recurrence of adverse incidents leading to unintentional harm. For this purpose, a detailed knowledge of the underlying causes of adverse incidents is essential. Root Cause Analysis (RCA) is a term used to describe a structured methodology for the retrospective investigation of adverse incidents, near misses and sentinel events [5,6], which was originally developed to analyse major industrial incidents [5,7]. The quality and efficacy of this process relies on consistent and meticulous incident reporting and analysis [1,8]. The concept of RCA was first introduced to the medical community in the mid 1990’s [9] and since then it has been playing an important role in improving patient care and safety in the United States, Great Britain and other countries [5].

The aim of our study was to determine how efficient the incident reporting process is in the Department of Surgery of Ninewells Hospital, Dundee, one of Europe’s largest teaching hospitals. We investigated the percentage of the recorded sentinel incidents that underwent RCA, whether any recommendations resulted from this process and what ratio of these recommendations were implemented in clinical practice between May 2004 and December 2009.

## Methods

The Adverse Incident Management (AIM) database for incident reporting and RCA was introduced in Ninewells hospital in May 2004. Adverse incidents at this unit are reported by staff members with the use of an online template form, which is available on the hospital intranet. An ordinal score with a range from 1 to 5 and a corresponding colour is allocated to each incident by the reporter, according to the severity of the incident as determined by a reference table (Table 1). Sentinel or Red incidents (score 4 or 5) include incidents that have potentially caused serious physical harm and/or deleterious financial effects, and thus gain priority in receiving an RCA. Adverse incidents are reviewed by staff members from various backgrounds and different levels of experience in performing RCA, i.e. surgeons, managers and senior nursing staff. Sentinel incidents are reviewed by a designated investigator and the outcome is reported to the executive management. A meeting with other members of the risk management team and the involved staff members may be deemed necessary during this process.

**Table 1:** The criteria used to determine the severity of incidents

		**Consequences**					
**Score**	**Descriptor**	**Objective**	**Cost**	**Physical harm**	**People affected**	**Schedule**	**Reputation**
**1 (Green)**	Negligible	Minimal impact, no service disruption	Minimal financial loss (<10 K)	No obvious harm or injury	None	Minimal	No interest to press. Internal
**2 (Green)**	Minor	Minimal impact on service provision	Moderate financial loss 10-50 K	First aid treatment. Non-permanent harm of up to 1 month	1-2	Increased level of care. Increased length of stay 1–7 days	Some public embarrassment. No damage to reputation or standing in the community
**3 (Amber)**	Moderate	Service objective partially achievable	Significant financial loss (50-100 K)	Medical treatment required. Semi- permanent harm up to 1 year	3-15	8-15 days. Pressure on service provision	Local adverse public embarrassment, leading to limited damage. Local MP interested on ME legal implication.
**4 (Red)**	Major	Significant impact on service provision	Major financial loss 100 K-1 million	Extensive injury with possible permanent harm	16-50	>15 days. Temporary service closure	National adverse publicity. May have caused loss of confidence in the organisation
**5 (Red)**	Catastrophic	Unable to function inability to fulfil corporate obligations	Significant financial loss (>1 million)	Death	>50	Extended service closure	Highly damaging international adverse publicity. Severe loss of public confidence. Court enforcement. Public accounts committee enquiry.

We retrospectively reviewed the recorded data for each sentinel incident reported by the Department of Surgery between May 2004 and December 2009. All adverse incident reports involving colorectal, breast, vascular, upper gastrointestinal and hepatobiliary surgical cases, as well as the ones from the Department of Urology and from the acute surgical admissions unit were included in our study. Our primary endpoints were the quality of the sentinel incident reports and the efficacy of RCA recommendations. Patient confidentiality was strictly protected at all stages of this study.

As a means to gain further information, we undertook interviews of members of staff that were involved with the reporting process and/or the reviewing procedure, including clinicians, managers and nursing staff. During the interviews, a brief description of the incident was provided as a reminder; the interviewees were then asked to provide additional information on the incident and the subsequent RCA, if one had been performed. They were also asked to state whether the suggested recommendations were, to their knowledge, implemented in clinical practice. The staff members beyond our reach were contacted via email or telephone.

In order to minimise bias, we interviewed a separate group of staff members that had not been involved in reporting/reviewing the incidents, on each of the wards that the sentinel incidents were reported from. The aforementioned endpoints for each sentinel incident were compared between the two groups of interviewees. The time period between each event and the subsequent interviews ranged from 2 months to 5 years.

## Results

**Figure 1:**
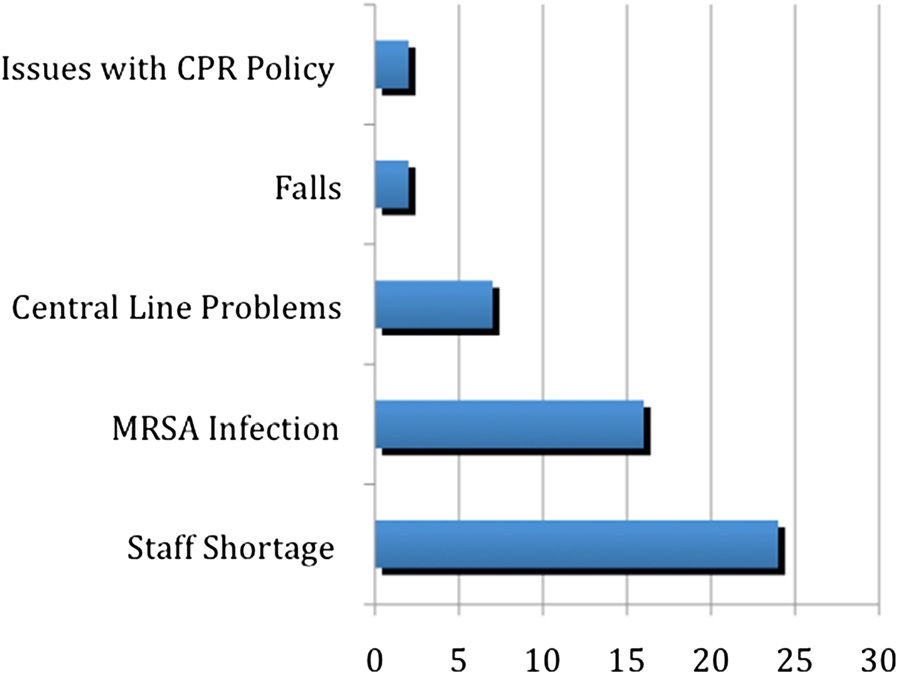
Most frequently reported surgical incidents.

Between May 2004, when the Adverse Incident Management (AIM) system was introduced in Ninewells Hospital, and December 2009 a total of 3,142 surgical incidents were reported. Eighty-one of the 3,142 incidents (2.58%) were characterised as sentinel or red, reaching an average of 1.2 sentinel incidents per month. Certain groups of incidents occurred more frequently (Figure 1). These included; 24 (29.6%) reports of staff shortage leading to major compromise in patient care, 16 (19.7%) reports of methicillin resistant staphylococcus aureus (MRSA) sepsis or issues with the related policy, 7 (8.6%) reports of central venous catheters complications, 2 (2.4%) reports of patient falls leading to debilitating morbidity and mortality and 2 (2.4%) reports of poor adherence to the local cardiopulmonary resuscitation (CPR) protocol. Based on the criteria of Table 1, 19 of the 81 incidents (23.4%) should not have been reported as red. This fact may have resulted from reporter bias in the selection of the incident severity score and perhaps from lack of awareness of the aforementioned criteria.

Deficiencies in the quality of the reports of sentinel incidents were also revealed; in 31 reports (38.2%) vital information was missing; in 12 cases (14.8%) the description of incidents was of poor quality and other reports had information stored in the wrong field of the template, rendering them incomprehensible or difficult to follow. A direct correlation was identified between the delay in reporting an incident and the deficiencies in the incident report. During the interview stage of our study, 43% of the interviewees who had been involved in either reporting an incident or the consequent RCA, were unable to recall the full details of respective incidents or did not recall them at all. This signifies the importance of prompt and concise reporting as soon as possible after the incident has occurred. We consider inadequate training in incident classification and reporting as an indisputable cause of the aforementioned deficiencies [10-12].

**Figure 2:**
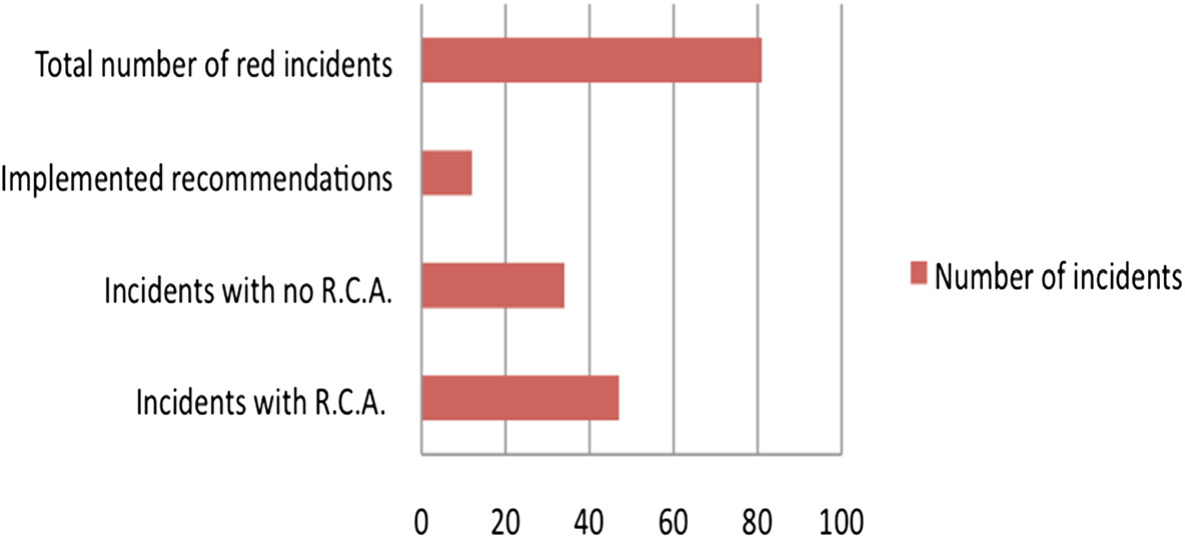
Proportion of red incidents that received RCA and had recommendations implemented in clinical practice.

**Figure 3:**
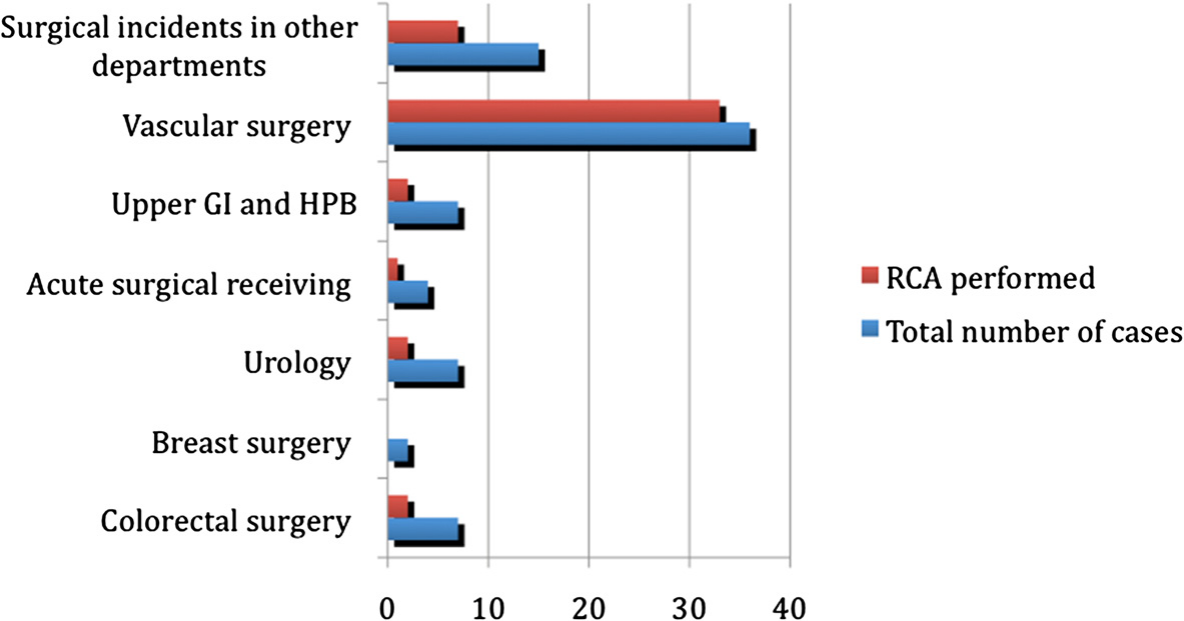
Breakdown of the source of sentinel incident reports and the ratio of the subsequent RCAs.

Of the 81 reported sentinel incidents 47 (58%) underwent RCA and only 12 (14.8%) had any recommendations implemented in clinical practice (Figures 2 &3). Repeated reports of similar incidents from the same source were identified, indicating inefficiency of the RCA process in particular cases. No substantial improvement was noted in the quality of RCAs or implementation of recommendations over time within the study period, and although the quality of RCA was variable amongst different surgical wards, we did not identify any major differences. Considering that sentinel incidents are prioritised for RCA, one could hypothesize that even fewer of the less serious adverse events (i.e. green and amber incidents) underwent RCA.

The efficacy of RCAs was variable depending on the nature of the incident. Adverse events that were deemed more likely to lead to patient harm, financial costs, or negative publicity received a more vigorous investigation. An example of an effective RCA with clinical implications was a case of iatrogenic small bowel perforation during a laparoscopic inguinal hernia repair (LIHR) performed by a surgical trainee, which was not identified intra-operatively. The patient subsequently required an urgent laparotomy and small bowel resection and made an uneventful recovery. In this case the RCA resulted in a substantial change in the local practice of LIHR introducing a minimum of one hundred supervised cases of LIHR before any trainee could perform this procedure unsupervised and a systematic small bowel examination before closure was implemented as a rule. However, many other reported sentinel incidents did not result in similar changes.

In order to further assess the quality of the implemented changes in clinical practice, we interviewed members of the nursing staff. We attempted to reduce bias by recruiting staff members that had not been involved in the incident reporting or RCA of incidents. We discovered that despite the positive feedback regarding some of the implemented changes, the overall impression was that in the majority of the cases only one aspect of a problem was being tackled instead of adopting broader improvements.

## Discussion

The aim of hospital care is to provide appropriate and safe service for patients. Nevertheless, up to 10% of acutely admitted patients are involved in adverse incidents and can therefore be unintentionally harmed [1-3]. Remarkably, up to two thirds of all adverse incidents take place in surgery [4], more than half of which may be preventable [4]. Prevention efforts depend on the detailed knowledge of the aetiologies of these events [13].

It is generally considered that errors are multifactorial in nature and that they occur due to a failure of the system rather than because of the failure of an individual in performing their task *per se*[14]. RCA is a method for retrospective analysis of systematically collected data regarding incidents with an aim to determine the main cause(s) of errors and to identify system or process weaknesses that contributed to or allowed an adverse event [9,15]. Reviewing adverse incidents with the involved members of staff is thought to allow a better understanding of the aetiologies of incidents by identifying the so called “root cause” [8]. The purpose of RCA is to answer three key questions: What was the adverse incident? How did it occur? Why did it occur? [9,15,16].

Literature defines root cause as the element that, if corrected, would prevent similar incidents from happening and assists in determining a management plan should the same or a similar incident occur [5,9]. However, based on the fact that almost all adverse incidents are multifaceted in nature, the terms root cause and RCA seem to be misnomers [7,8,16]. In addition, RCA as a term implies that the sole aim of investigation is to discover what caused an adverse incident, whereas the main principle behind this process is to reveal inadequacies in the healthcare system and look at the broader picture [16]. Nevertheless, it is readily used in the English literature and we have not broken this tradition in our report.

Considering the complex nature of the aetiologies of adverse incidents, when an adverse incident occurs, it would be inappropriate to place blame on individuals. RCA is a method whereby focus is placed on mutual learning and in-depth discussions regarding the incident in a non-threatening and non-blaming environment [7,12,17]. There is evidence that if performed correctly, RCA is of great value in identifying root causes, facilitating incident management and error reduction, thereby optimising patient care and safety in a healthcare system [10,15]. It is therefore an extremely valuable tool in disposal of modern healthcare.

Institutions perform RCA using either a “team-based” or “investigator led” methods. There are certain merits to the team-based approach compared to the investigator led method. In the latter method, an investigator assembles the relevant information relating to the incident and reports to the management team so that the relevant changes can be implemented. However, in many centres it is now felt that a team of investigators with a wide spectrum of skill and backgrounds e.g. risk managers and clinicians should perform RCA in particular in relation to the serious adverse incidents using a predetermined protocol. This would allow for a more effective and thorough analysis of incidents [18], as supported by our findings.

According to Reason’s model of human errors [14], the team of investigators should take 3 important factors into consideration for the investigation: Care management problems (i.e. actions taken by members of staff that is thought to have led to the occurrence of the incident), the clinical context of the incident and any factors contributing to its occurrence [12,16]. A chronological flow chart of the incident is then drawn, so as to tease out where the system failed. The team then analyses the incident, identifies possible aetiologies, and devises plans to address any issues, aiming to ensure that a similar incident will not occur. The incident reporter is then contacted with a course of action. After the implementation of the plan, feedback is provided to ensure the effectiveness of recommendations [12,16] . RCA must be considered as an important duty, which would require sufficient time and active input by the members of the team [12].

Despite the benefits that RCA offers, certain limitations exist. Although it aims to answer the 3 important questions mentioned earlier, in many circumstances it does not seem to reduce the risk of an adverse incident recurring at a later date [15]. Therefore adverse incidents frequently recur despite time and resource consuming RCAs [15]. It often suffers from hindsight bias; adverse incidents are perceived to be more predictable after they have taken place, whereas in reality this is usually not the case [9,19].

RCAs are time and resource consuming [15]. Many incidents are multifactorial in nature and determining a root cause is a great challenge [9,13]. Other reported limitations of this system include: uncooperative colleagues, inter-professional differences, poor software performance, lack of structured reporting and unsupportive management [10]. Furthermore, it is very difficult to follow-up the outcomes and recommendations achieved from RCA. Experience has shown that recommendations are not always adhered to in clinical practice and incidents repeat themselves despite good quality RCA and recommendations [15]. In support of this argument, our study revealed repeated similar reports from the same sources. RCA should result in recommendations, which need to be implemented in clinical practice, in order to be cost-effective. For this purpose, active support from the management is required and the necessary resources need to be provided; otherwise the likelihood of improvement is limited [12].

Reporting incidents can be challenging. Staff members often have concerns about personally admitting a mistake and worry about penalty or litigation [1]. Moreover, it is often difficult to categorise incidents according to their severity, and they are often incorrectly, incompletely and/or incoherently recorded on the database [20]. In our study more than 50% of the reported incidents were of inadequate quality. Certain centres have provided Safety Improvement Programmes (SIP) for training staff in incident reporting with a focus to improve their RCA results. The SIP covers topics such as: incident identification and prioritisation, systematic notification of incidents to the individuals concerned, investigation using the RCA approach, actions required regarding recommendations, feedback of the collected data to the system and appropriate discussion regarding sentinel incidents. Evidence in the literature suggests that SIP improves the quality of incident management thereby leading to error reduction [10-12,21]. Reports from U.K. hospitals, included exemplary practice of RCA in only 2 of the 7 centres studied (29%), less rigorous practice in 3 (43%) and scanty practice in the other 2 units [21]. The reports showed a definite correlation between training and the quality of RCA [21,22].

Our study demonstrates certain deficiencies of the quality of incident reports, the ratio of sentinel incidents that underwent RCA and the implementation of changes in clinical practice. We discovered that a significant number of incident reports were of poor quality, a fact that can adversely affect the outcome of a subsequent RCA. To improve the quality of incident reports, we recommend targeted formal training of staff members in incident reporting. Sentinel incidents should be reported shortly after their occurrence which, would in-turn allow for an early RCA, and the potential recommendations would hopefully prevent the recurrence of similar incidents as early as it may be possible. Hospital management should take a more active role in ensuring that any recommendations are implemented in clinical practice. The decision for an in-depth analysis of sentinel incidents was variable and depended on the subjective judgement of a designated reviewer. A predetermined protocol for the analysis of sentinel incidents would certainly contribute to improved results. Furthermore, the inconsistency of the incident report reviewers in terms of training and experience in RCA may have led to discrepancy in the quality of RCAs, and may have affected the implementation of recommendations.

## Conclusion

In conclusion, RCA is an important component of risk management and clinical governance that aims to prevent patient harm, with enormous potential benefits. It does however rely on the good quality of adverse incident reporting. RCAs are cost-effective only if incidents are thoroughly analysed in order for recommendations to be made, and subsequently to be implemented in clinical practice. Our study highlights the importance of prompt and good quality incident reporting in facilitating the analysis of adverse incidents. A predetermined protocol for the analysis of sentinel incidents by assigned investigators would contribute to objective and more efficient RCAs.

We consider regular formal staff training and vigorous reviews of this system necessary. Furthermore, sufficient support and resources are required for the implementation of RCA recommendations in clinical practice.

## Competing interests

There has been no competing interest with regards to this article.

## Authors’ contributions

MK: Main author, data collector and interviewer. CS: Manuscript drafting, data interpretation, critical analysis of the data. KB: Manuscript drafting, critical analysis of the manuscript. AA: Project supervisor, critical analysis of the manuscript. All authors read and approved the final manuscript.

## Authors’ information

1. Maziar Khorsandi, MBChB, MRCS, BMSc, Senior House Officer, Department of Cardiothoracic Surgery, Royal Infirmary of Edinburgh, Edinburgh

2. Christos Skouras, MD, MRCS, Specialist Registrar, Department of Surgery, Royal Infirmary of Edinburgh, Edinburgh

3. Kevin Beatson, MBChB, Senior House Officer, Department of Orthopaedic Surgery, Royal Infirmary of Edinburgh, Edinburgh

4. Afshin Alijani, MBChB, FRCS, PhD, Consultant Surgeon - Chairman of the Clinical Governance and Safety Committee, , Department of Surgery, Ninewells hospital, Dundee
